# Involvement of Crosstalk between Oct4 and Meis1a in Neural Cell Fate Decision

**DOI:** 10.1371/journal.pone.0056997

**Published:** 2013-02-25

**Authors:** Takeyuki Yamada, Yumiko Urano-Tashiro, Saori Tanaka, Hirotada Akiyama, Fumio Tashiro

**Affiliations:** Department of Biological Science and Technology, Faculty of Industrial Science and Technology, Tokyo University of Science, Noda-shi, Chiba, Japan; Kanazawa University, Japan

## Abstract

Oct4 plays a critical role both in maintaining pluripotency and the cell fate decision of embryonic stem (ES) cells. Nonetheless, in the determination of the neuroectoderm (NE) from ES cells, the detailed regulation mechanism of the *Oct4* gene expression is poorly understood. Here, we report that crosstalk between Oct4 and Meis1a, a Pbx-related homeobox protein, is required for neural differentiation of mouse P19 embryonic carcinoma (EC) cells induced by retinoic acid (RA). During neural differentiation, Oct4 expression was transiently enhanced during 6–12 h of RA addition and subsequently disappeared within 48 h. Coinciding with up-regulation of Oct4 expression, the induction of Meis1a expression was initiated and reached a plateau at 48 h, suggesting that transiently induced Oct4 activates *Meis1a* expression and the up-regulated Meis1a then suppresses *Oct4* expression. Chromatin immunoprecipitation (ChIP) and luciferase reporter analysis showed that Oct4 enhanced *Meis1a* expression via direct binding to the *Meis1* promoter accompanying histone H3 acetylation and appearance of 5-hydoxymethylcytosine (5hmC), while Meis1a suppressed *Oct4* expression via direct association with the *Oct4* promoter together with histone deacetylase 1 (HDAC1). Furthermore, ectopic *Meis1a* expression promoted neural differentiation via formation of large neurospheres that expressed Nestin, GLAST, BLBP and Sox1 as neural stem cell (NSC)/neural progenitor markers, whereas its down-regulation generated small neurospheres and repressed neural differentiation. Thus, these results imply that crosstalk between Oct4 and Meis1a on mutual gene expressions is essential for the determination of NE from EC cells.

## Introduction

Cell fate decisions are fundamental for development, but we do not know how core pluripotency circuit genes, including *Oct4, Sox2, Nanog, Klf4/5,* and *Tbx3*, reorganize transition from a pluripotent to a differentiated cell state [1]. Oct4, encoded by the *Pou5f1* gene, belongs to the POU-homeodomain family of transcription factors and binds to an octamer motif, ATGCAAAT [2]. Oct4 is the main regulator of pluripotency during the earliest stages of vertebrate development and its expression is confined to pluripotent cells of the developing embryo, including epiblast and primordial germ cells, as well as their *in vitro* counterparts, embryonic stem (ES) and embryonic germ cells [3], [4]. It has been demonstrated that the generation of induced pluripotent stem (iPS) cells from mouse and human fibroblasts are achieved by introducing four factors, Oct4, Sox2, Klf4, and c-Myc [5], [6]. Kim *et al.* also reported that Oct4 is sufficient to generate iPS cells from adult mouse neural stem cells [7]. Critical expression levels of *Oct4* mRNA in ES and embryonic carcinoma (EC) cell lines such as P19 and F9 cells are rapidly down-regulated by differentiation induced with retinoic acid (RA) 8,9. In mouse ES cells, a less than twofold increase in expression causes differentiation into the primitive endoderm and mesendoderm (ME), whereas a reduction to that less than a normal level triggers differentiation into the trophectoderm [10]. Targeted disruption of the *Oct4* gene in mice results in embryonic death at the blastocyst stage, and compacted morula cells differentiate only into the trophectoderm [11]. An Oct4 expression level of 50–150% of the endogenous amount in ES cells is permissive for self-renewal and the maintenance of pluripotency [12]. Thus, the expression level of Oct4 is crucial not only for the maintenance of pluripotency but also for early cell differentiation decisions [10].

Previous studies have shown that many transcription factors including SF-1, GCNF, RAR/RXR, COUP-TFI/II, LRH-1, CDX2, and the Oct4/Sox2 complex regulate *Oct4* gene expression via binding to its proximal enhancer and promoter and distal enhancer during ES cell differentiation into progenitors of the ME or trophectoderm [13]. However, it remains to be clarified as to how ES cells leave the pluripotent state and choose the neuroectoderm (NE). Shimozaki *et al.* have reported that sustained exogenous Oct4 expression in ES cells cultured without serum and LIF caused accelerated differentiation to NE-like cells expressing Sox2, Otx1, and Emx2 and subsequently differentiated into neurons [14]. Recently, Thomson *et al.* have shown that Oct4 suppresses NE differentiation from ES cells and promotes ME differentiation, while Sox2 inhibits ME and promotes NE differentiation [1]. These findings indicate that differentiation signals continuously and asymmetrically modulate Oct4 and Sox2 protein levels, altering their binding pattern in the genome, leading to a cell fate decision. On the other hand, Archer *et al.* have reported that overexpressed Oct91, the Xenopus homolog Oct4, cooperates with Sox2 to maintain neural progenitor marker expression, and knockdown of Oct91 inhibits neural induction driven by either Sox2 or Sox3 [15]. Thus, the precise function of Oct4 and how its expression is regulated in the neural fate decision are not fully understood.

Meis1 (myeloid ecotropic viral insertion site1) was identified in the leukemic cells of BXH-2 mice [16]. Three genes constitute the mammalian Meis family with *Meis1* transcripts alternatively spliced to yield multiple isoforms [16]. Moreover, *Meis*-related genes *Prep1* and *Prep2* (for PBX regulatory protein) have also been identified [17]. Meis or Prep proteins are required for the PBX-Hox complex to exert transcriptional control [17]. Meis family proteins cooperate with PBX and Hox for hindbrain patterning in Xenopus, zebrafish, and mice [18]–[20]. In the developing olfactory epithelium (OE), slow dividing, self-renewing neural stem cells express a high level of Meis and reside primarily in the lateral OE, whereas rapidly dividing neurogenic precursors express high levels of *Sox2* and *Ascl1*, a neurogenic bHLH transcription factor, and reside mostly in the medial OE [21]. These identities have been established in part by a transcriptional network involving Meis1 activity, Sox2 doses, and Ascl1 expression that regulates the progression from a multipotent precursor to transit an amplifying neuronal progenitor and post-mitotic neurons such as the olfactory receptor, and vomeronasal and gonadotropin releasing neurons [21]. In addition, in Meis1-deficient embryos, definitive myeloerythroid lineages are present, but the total number of colony forming cells is dramatically reduced [22]. Thus, Meis1 activity is required for maintenance of the multipotencies of neural and hematopoietic stem cells. However, little is known about the precise function and regulation mechanism of the *Meis1* gene.

In this study, using the retinoic acid (RA)-induced mouse P19 EC cell neural differentiation system [23], we showed the possibility that up-regulated expression of Oct4 within 12 h of the immediate-early stages promotes *Meis1a* gene expression, whereas increased Meis1a suppresses *Oct4* gene expression. Moreover, ectopic expression of *Meis1a* caused the down-regulation of Oct4 and augmented neural differentiation via formation of large neurospheres in which neural stem cell (NSC)/neural progenitor markers were expressed. Thus, reciprocal regulation between Oct4 and Meis1a on mutual gene expressions is crucial for neural fate choice.

## Materials and Methods

### Cell Culture and Animals

P19 cells were obtained from the American Type Culture Collection (Manassas, VA). To induce neural differentiation, 1×10^6^ aggregated P19 cells were cultured in 10-cm bacteriological grade dishes in 10 ml of α-minimal essential medium (α-MEM) containing 10% fetal bovine serum (FBS) and 5×10^−7^ M all-*trans*-RA (Sigma-Aldrich, St. Louis, MO) for 4 days. Cell aggregates were suspended by mild pipetting and transferred to tissue culture dishes. Cells were cultured in RA-free α-MEM containing 10% FBS for an additional 3 days to induce β-tubulin (III)-positive neurons and for 7 days to induce glial fibrillary acidic protein (GFAP) and S100β-positive astroglial cells.

ICR mice were purchased from Charles River Japan (Kanagawa, Japan).

### Ethics Statement

Mouse care and handling conformed to the National Institute of Health Guidelines for Animal Research. Experimental protocols were approved by the Institutional Animal Care and Use Committee (IACUC protocol #N12018 to FT).

### Gene Expression Analysis by RT-PCR and Northern Blotting

RT-PCR and Northern blotting were performed as described previously [23]. The following primer sets were used for RT-PCR: *Meis1a/b* (5′-primer; 5′-tgc ccg gag aag aat agt gca g-3′, 3′-primer; 5′-ctt ggg tat aac tcg gct gtc c-3′), *Oct4* (5′-primer; 5′-cga gga gtc cca gga tat ga-3′, 3′-primer; 5′-gtt cca cct cac acg gtt ct-3′), *Sox2* (5′-primer; 5′-aac tat tct ccg cca gat ctc c-3′, 3′-primer; 5′-aat ctc tcc cct tct cca gtt c-3′), *Pax6* (5′-primer; 5′-tgg gat ccg gag gct gcc aac-3′, 3′-primer; 5′-atc gtt ggt aca gac ccc ctc gg-3′), and *P0* (5′-primer; 5′-cag ctc tgg aga aac tgc tg-3′, 3′-primer; 5′-gtg tac tca gtc tcc aca ga-3′). An *Apa*I-*Sac*I fragment of pcDNA3-EF1-α-*Meis1a* was used for Northern blotting as a probe [23].

### Western Blotting (WB)

Cells were lysed with SDS sample buffer (62.5 mM Tris-HCl, pH6.8, 2% SDS, 10% glycerol, and 5% DTT) and analyzed by WB as described previously [23]. The following antibodies were used: anti-Meis1 (1∶500, Santa Cruz Biotechnology, Santa Cruz, CA), anti-Oct4 (1∶1000, Santa Cruz Biotechnology), anti-β-tubulin (III) (1∶1000, Sigma-Aldrich), anti-GFAP (1∶2000, Sigma-Aldrich), anti-S100β (1∶1000, Santa-Cruz Biotechnology), anti-Nestin (1∶3000, kindly provided by Y. Tomooka [24]), anti-GLAST (1∶500, Abnova, Taipei, Taiwan), anti-brain lipid-binding protein (BLBP; 1∶1000, Millipore, Billerica, MA), anti-Sox1 (1∶1000, Santa Cruz Biotechnology), anti-Sox2 (1∶1000, Abcam, Cambridge, UK), anti-Pax6 (1∶1000, Abcam), and anti-β-Actin (1∶1000, Santa Cruz Biotechnology).

### Immunocytochemical Analysis

P19 cells were fixed with 4% paraformaldehyde and stained with anti-β-tubulin (III) and anti-GFAP antibodies described above, followed by anti-mouse IgG conjugated with Cy3 (Jackson Immuno Research, West Grove, PA). Nuclei were stained with Hoechst 33258. Cells were observed under a fluorescence microscope (Axiovert 200; Carl Zeiss, Oberkochen, Germany).

### Mifepristone (MIF)-controlled RNA Expression

MIF-controlled sense/antisense *Meis1a* RNA expression in P19 cells was performed using the GeneSwitch™ System (Invitrogen, Carlsbad, CA). At the first step, P19 cells were transfected with pSwitch by lipofection with Lipofectamine and Plus Reagents (Invitrogen) and were cultured in the presence of 300 µg/ml hygromycin (Wako, Tokyo, Japan) for selection. Selected P19 cells were transfected with pGene/V5-His B/sense *Meis1a* or antisense *Meis1a*, which were constructed by the insertion of PCR-amplified *Meis1a* cDNA (as described below) into pGene/V5-His (Invitrogen) in sense and antisense directions, respectively, and were selected in the presence of 50 µg/ml zeocin (Invitrogen). Selected pGene/V5/sense and antisense *Meis1a*-intrduced P19 cells were designated S-Meis1a or AS-Meis1a. Upon the addition of 1×10^−7^ M MIF (Invitrogen), these transfectants efficiently expressed sense/antisense *Meis1a* RNAs.

### Construction of Transient Expression Vectors for *Meis1a* and *Oct4*

pcDNA3-EF1-α-*Meis1a* and pcDNA3-EF1-α-*Oct4* were constructed by the insertion of PCR-amplified *Meis1a* cDNA (5′-primer; 5′-gaa ttc gaa ggg agc cag aga g-3′, 3′-primer; 5′-gtc gac cga gat cag tca cca t-3′) and *Oct4* cDNA (5′-primer; 5′-acc gaa ttc ccc atg gct gga c-3′, 3′-primer; 5′-aaa gcg gcc gcg ctc ctg atc aa-3′) into pcDNA3-EF1-α, respectively [23].

### Luciferase Reporter Assay

Luciferase reporter plasmids *Meis1*(−926)-Luc, *Meis1*(−355)-Luc, and *Meis1*(−92)-Luc were constructed by the insertion of PCR-amplified mouse *Meis1* promoter regions (−926 to +47, −355 to +47 and −92 to +47) into pGL4.10 (Promega, Madison, WI), respectively. Primer sets used were as follows: −926; 5′-primer; 5′-ggg gta ccc tac gtc cac ttc tga c-3′, −355; 5′-primer; 5′-ggg cct act tgc tgc agc cca a-3′, −92; 5′-primer; 5′-ggg gta ccg aga gga act gat tag g-3′, +47 common 3′-primer; 5′-ggg aga tct gag gtt gtc aac gtg g-3′. Reporter plasmids *Oct4*(−1059)-Luc, *Oct4*(−698)-Luc, *Oct4*(−506)-Luc, and *Oct4*(−254)-Luc were also constructed by the insertion of PCR-amplified mouse *Oct4* promoter regions (−1059 to +255, −698 to +255, −506 to +255 and −254 to +255) into pGL4.10, respectively. Primer sets used were as follows: −1059; 5′-primer; 5′-aag gga agc agg gta cct cca tct ga-3′, −698; 5′-primer; 5′-tga ggt acc agg ccc cgg cct taa-3′, −506; 5′-primer; 5′-gtg tgg tac ctc taa act ctg gag g-3′, −254; 5′-primer; 5′-tgg ggt acc cga gca act ggt ttg-3′, +225 common 3′-primer; 5′-aca tgg gga gat ctc caa tac ctc tg-3′).

In the case of *Meis1* promoter analysis, for each transfection, P19 cells (5×10^3^ cells/well of 96-well dish) were transfected with 30 ng Meis1-Luc reporter, 15 ng pcDNA3-EF1-α-*Oct4,* and 3 ng Renilla luciferase expression vector pGL4.75 (Promega) as an internal control by lipofection. After 24 h, luciferase activities were assayed using the Dual-Luciferase Reporter Assay System (Promega). The *Oct4* promoter was also analyzed in the same conditions except for 30 ng *Oct4*-Luc and 60 ng pcDNA3-EF1-α-*Meis1a*.

### Chromatin Immunoprecipitation (ChIP) Analysis

The ChIP assay was carried out as described previously [23]. Briefly, aggregated P19 cells treated with RA for 0, 6, and 12 h were cross-linked with 1% formaldehyde. Cell extracts were sonicated to shear genomic chromatin and were immunoprecipitated with anti-Oct4, anti-Meis1, anti-acetylated histone H3 (AcH3; Millipore), and anti-histone deacetylase 1 (HDAC1; Santa Cruz Biotechnology), anti-5-hydroxymethylcytosine (5hmC; Active Motif, Carlsbad, CA) and anti-5-methylcytosine (5mC; Active Motif) antibodies. Primer sets used for PCR were as follows; *Meis1* promoter region (−360 to −67): 5′-primer; 5′-tac ttg ctg cag ccc aat gca t-3′, 3′-primer; 5′-cct tga atc agt cct aat tcc t-3′, *Oct4* promoter region (−1062 to −778): 5′-primer; 5′-agc agg gta tct cca tct gag g-3′, 3′-primer; 5′-ggg agg tgg gta gag aga aga a-3′.

### Neurosphere Formation

Aggregated S-Meis1a and AS-Meis1a cells were cultured with RA in the presence or absence of MIF for 4 days. Images of non-overlapping twenty fields/sample were selected under a phase-contrast microscope and more than 400 spheres (>50 µm in diameter) were counted.

### Statistical Analysis

All data were expressed as mean ± SE of the indicated number of experiments. Comparisons of data were carried out by the Student’s *t*-test. Differences were considered significant at *p*<0.05. The software package KaleidaGraph 3.6 (Synergy Software, Reading, PA) was used for statistical analysis.

## Results

### Reciprocal Relationship between Meis1a and Oct4 Expressions during Neural Differentiation

**Figure 1:**
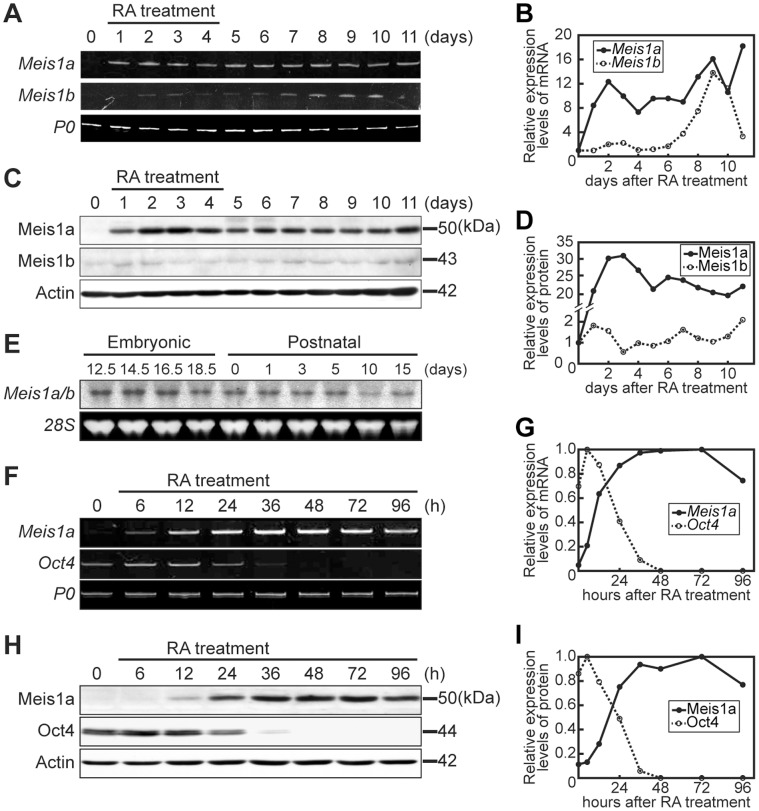
Induction of Meis1a/b expressions during neural differentiation of RA-primed P19 cells. Aggregated P19 cells were treated with 5×10^−7^ M RA for various times and the expression levels of Meis1a/b mRNAs and proteins were analyzed by RT-PCR and WB with the anti-Meis1 antibody, respectively. (**A**) Expression patterns of *Meis1a* and *Meis1b* mRNAs during neural differentiation. (**B**) Quantification of the expression levels of *Meis1a* and *Meis1b* mRNAs indicated in **A**. (**C**) Expression patterns of Meis1a and Meis1b proteins during neural differentiation. (**D**) Quantification of the expression levels of Meis1a and Meis1b proteins indicated in **C**. (**E**) Expression levels of *Meis1a/b* mRNAs in mouse fetal brain. Total RNAs from the developing brain were analyzed by Northern blotting. (**F**) Detailed expression patterns of *Meis1a* and *Oct4* mRNAs during neural differentiation. (**G**) Quantification of the expression levels of *Meis1a* and *Oct4* mRNAs indicated in **F**. (**H**) Detailed expression patterns of Meis1a and Oct4 proteins during neural differentiation. (**I**) Quantification of the expression levels of Meis1a and Oct4 proteins indicated in **H**. *Ribosomal large subunit protein P0* mRNA, β-actin and 28S ribosomal RNA were used as internal controls.

Meis1 has two splicing variants, Meis1a and Meis1b, whose C terminal transcription regulatory regions are different from each other [25]. Based on this fact, we examined whether Meis1a and Meis1b expressions were induced during neural differentiation of RA-primed P19 cells. *Meis1a* mRNA and protein expressions were substantially induced in a similar manner within 1 day of RA addition and these high expression levels were sustained for up to 11 days (Fig. 1A–D). On the other hand, *Meis1b* mRNA and protein expressions were finally initiated after 7 days. Furthermore, Northern blot analysis indicated that *Meis1a/b* transcripts were expressed in fetal and postnatal mouse brains and maximal expression was observed embryonic 14.5 days, when neurogenesis is most active (Fig. 1E). We previously reported that the neural cell fate decision in P19 cells is carried out during 2 days of RA addition, suggesting that Meis1a, but not Meis1b, is deeply involved in neural differentiation. Therefore, more detailed expression patterns of *Meis1a* mRNA and protein were analyzed together with those of *Oct4*. Meis1a expression was initiated within 12 h of RA addition, when Oct4 was transiently up-regulated (Fig. 1F–I). On the other hand, Oct4 expression disappeared after 36 h in anti-parallel to the maximal level of Meis1a expression. The characteristic up-regulation of Oct4 within 12 h of the immediate-early stages of neural differentiation of RA-primed P19 cells was consistent with previous observations [23]. Thus, these results suggest that Meis1a takes part in neural differentiation via some interaction with Oct4.

### Meis1a Promotes Neural Differentiation

**Figure 2:**
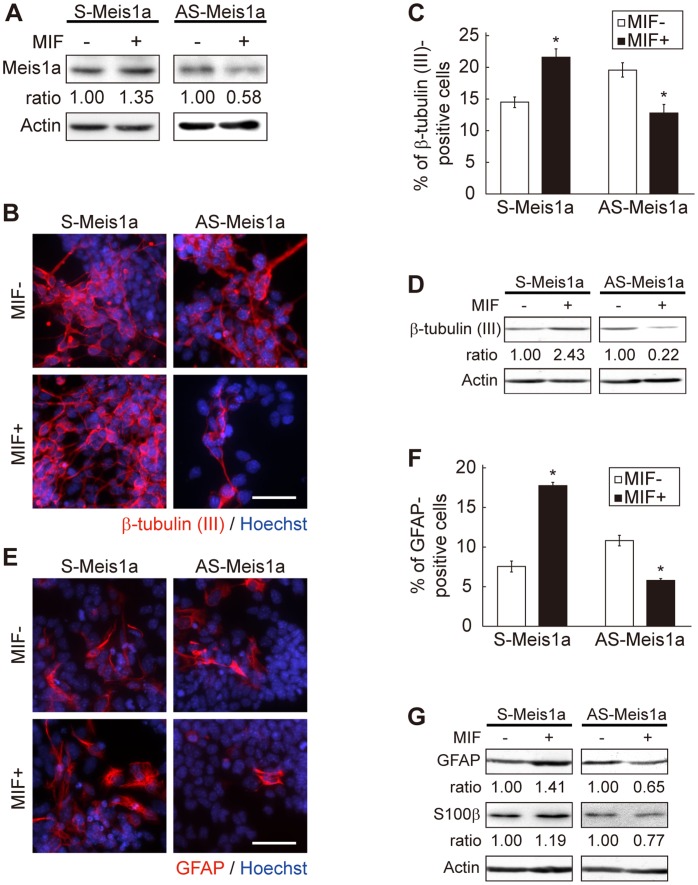
Involvement of Meis1a in neural differentiation of RA-primed P19 cells. (**A**) Expression levels of Meis1a in S-Meis1a and AS-Meis1a cells with or without MIF. (**B**) Functional analysis of Meis1a in neuronal differentiation. Aggregated S-Meis1a and AS-Meis1a cells were treated with RA in the presence or absence of MIF for 4 days, additionally cultured in RA-free medium for 3 days, and stained with anti-β-tubulin (III) antibody, followed by Cy3-conjugated anti-mouse IgG antibody. Nuclei were stained with Hoechst 33258. *Scale bar* = 100 µm. (**C**) Quantification of the effect of Meis1a on neuronal differentiation indicated in **B**. (**D**) Effect of Meis1a on β-tubulin (III) expression. Differentiated S-Meis1a and AS-Meis1a cells with or without MIF were lysed and analyzed by WB with anti-β-tubulin (III) antibody. (**E**) Effect of Meis1a on GFAP-positive astrocyte differentiation. RA-treated S-Meis1a and AS-Meis1a cells were additionally cultured for 7 days and then stained with anti-GFAP antibody. (**F**) Quantification of the effects of Meis1a on astrocyte differentiation indicated in **E**. (**G**) Effect of Meis1a on GFAP and S100β expressions. Differentiated S-Meis1a and AS-Meis1a cells with or without MIF were analyzed by WB with the anti-GFAP and anti-S100β antibodies. *p<0.005 significantly different from MIF(−) control cells. *n = *3.

To investigate the function of Meis1a in neural differentiation, we established the P19 subcell lines S-Meis1a and AS-Meis1a, in which exogenous expression of sense and antisense *Meis1a* RNAs could be initiated by the addition of MIF, respectively. After 24 h of MIF addition, Meis1a expression levels in S-Meis1a and AS-Meis1a cells were estimated to be 1.35 and 0.58-fold of those in MIF-non added control cells, respectively (Fig. 2A).

Using these cells, we investigated the effect of *Meis1a* on RA-primed neuronal differentiation by immunocytochemical analysis with the anti-β tubulin (III) antibody. In the presence of MIF, β-tubulin (III)-positive neurons were more highly induced in S-Meis1a cells than that in the MIF-untreated control cells, while neuronal differentiation in AS-Meis1a cells was suppressed (Fig. 2B, C). The effect of *Meis1a* on astrocyte differentiation was also examined with immunocytochemical analysis with the anti-GFAP antibody. In the presence of MIF, the differentiation of RA-primed S-Meis1a cells to GFAP-positive astrocytes was enhanced as compared with that in MIF-untreated controls, whereas astrocyte differentiation in AS-Meis1a cells was significantly decreased (Fig. 2E, F).

We further analyzed the effect of ectopic expression of *Meis1a* on neural differentiation of P19 cells by WB. In S-Meis1a cells, expression levels of the neuronal marker β-tubulin (III), and astroglial markers GFAP and S100β were increased by the addition of MIF, whereas these markers were decreased in AS-Meis1a cells in the presence of MIF (Fig. 2D, G), coinciding with observations by immunocytochemical analysis (Fig. 2B, C, E, F). Thus, it seems that Meis1a is implicated in both neuronal and astrocyte differentiations.

### Oct4 Activates Meis1 Promoter Activity

**Figure 3:**
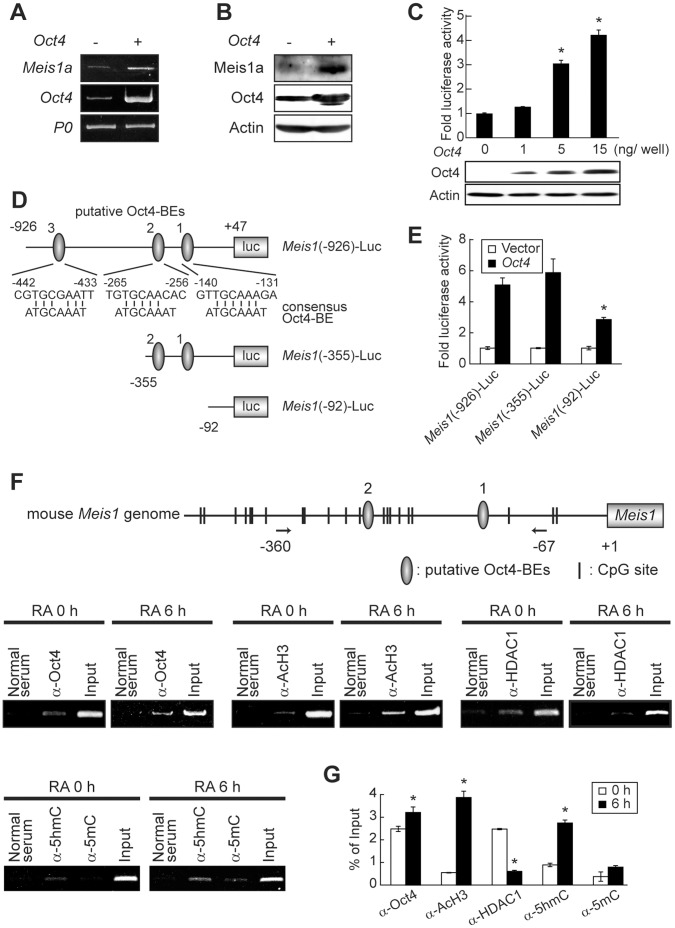
Oct4 activates Meis1a expression. Monolayer-cultured P19 cells were transfected with the pcDNA3-EF1-α-*Oct4* expression vector and after 24 h *Meis1a* and *Oct4* mRNAs and proteins were analyzed by RT-PCR (**A**) and WB (**B**), respectively. (**C**) Stimulatory effect of Oct4 on *Meis1* promoter activity. P19 cells were transfected with *Meis1*(−926)-Luc and various amounts of pcDNA3-EF1-α-*Oct4*. After 24 h, luciferase activities and expression levels of Oct4 were analyzed. *p<0.001 significantly different from vacant vector introduced control cells. (**D**) Schematic presentation of *Meis1* promoter-inserted luciferase reporter vectors. *Meis1*(−926)-Luc possesses three putative Oct4-BEs. (**E**) Functional analysis of Oct4-BEs. P19 cells were transfected with indicated *Meis1*-Luc vectors and pcDNA3-EF1-α-*Oct4.* After 24 h, luciferase activities were assayed. *p<0.001 significantly different from *Meis1*(−926)-Luc and *Meis1*(−335)-Luc. *n = *3. (**F**) Association of Oct4, HDAC1, AcH3, 5mC and 5hmC with the *Meis1* promoter. Genomic chromatin fragments from RA-treated aggregation form of P19 cells for 0 and 6 h were immunoprecipitated with the indicated antibodies and then DNAs were extracted. PCR was carried out using the primer set covering the −360 to −67 region of Meis1 promoter, in which 12 CpG sites exist. Aliquots of 10% antibody-untreated DNA samples were used for input DNA. (**G**) Quantification of the *Meis1* promoter-bound Oct4, HDAC1, AcH3, 5mC and 5hmC indicated in **F**.

The characteristic expression patterns of Oct4 and Meis1a in neural differentiation suggested the possible crosstalk between Oct4 (Fig. 1F–I). To examine whether Oct4 induces *Meia1a* expression, P19 cells were transfected with the *Oct4* expression vector and after 24 h expression levels of the *Meis1a* transcript and protein were analyzed. *Meis1a* mRNA and protein expression levels were significantly higher by the ectopic expression of *Oct4* than those in vacant vector-introduced cells (Fig. 3A, B). To further analyze the effect of Oct4 on *Meis1a* transcription, we constructed luciferase reporter plasmids, which were inserted at −926 to +47, −335 to +47, and −92 to +47 promoter regions of *Mies1* (relative to exon 1 transcription start site at +1) and designated them as *Meis1*(−926)-Luc, *Meis1*(−335)-Luc, and *Meis1*(−92)-Luc, respectively. In P19 cells, *Meis1*(−926)-Luc activity, which possesses three putative Oct4 binding elements (Oct4-BEs; consensus Oct4-BE; ATGCAAAT), was significantly stimulated by Oct4 in a dose-dependent manner (Fig. 3C). However, *Meis1*(−92)-Luc activity, in which Oct4-BE was deleted, was robustly lower than those of *Meis1*(−926)-Luc and *Meis1*(−335)-Luc (Fig. 3D, E). In addition, deletion of putative Oct4-BE3 did not affect luciferase activity, indicating that this promoter region is not essential for Oct4-dependent *Meis1* expression.

Using the ChIP assay, we analyzed whether Oct4 protein binds to putative Oct4-BEs1/2 in the *Meis1* promoter during neural differentiation. Before RA addition, putative Oct4-BEs1/2-bound Oct4 was detected together with HDAC1, a component of transcripitional repressor complexes [26] and the binding level of Oct4 was slightly lower than that form the cells treated with RA for 6 h, when Oct4 expression was temporally up-regulated and Meis1a expression was just initiated (Fig. 3F, G). Furthermore, during neural differentiation AcH3 and 5hmC as a possible marker of active chromatin were detected in this region, in which 12 CpG sites exist [27]. On the contrary, 5mC level in this region in the undifferentiated state was not significantly different from that in the differentiating cells. Although the detailed regulation mechanism of 5mC hydroxylation in CpG sites during neural differentiation is presently unknown, these results imply at least in part that Oct4-associated repressor complex containing HDAC1 could be converted to activator complex containing histone acetyltransferase (HAT) by the neural differentiation signals such as cell aggregation and RA.

### Meis1a Suppresses *Oct4* Expression

**Figure 4:**
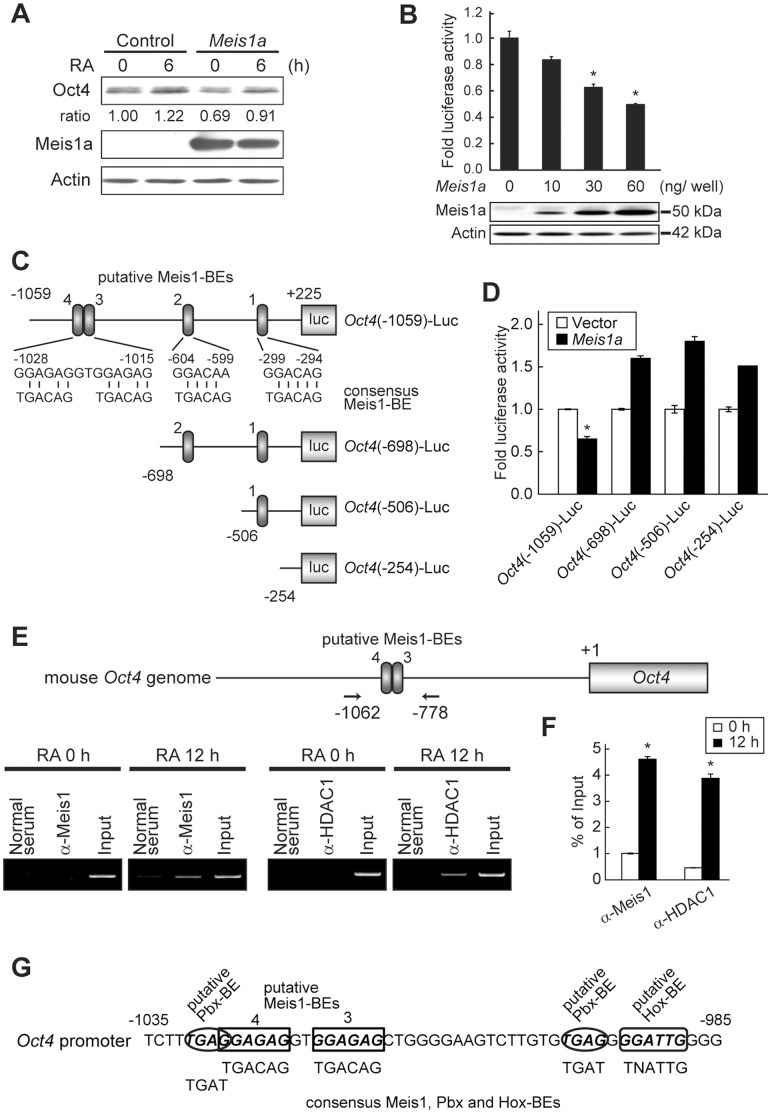
Meis1a suppresses Oct4 expression. (**A**) Reduction of Oct4 by ectopic expression of *Meis1a* RNA. P19 cells were transfected with the vacant vector or pcDNA3-EF1-α-*Oct4* and after 24 h, these aggregated cells were treated with RA for 0 and 6 h. Thereafter, expression levels of Oct4 and Meis1a proteins were analyzed by WB with anti-Oct4 and anti-Meis1 antibodies. (**B**) The suppressive effect of Meis1a on *Oct4* promoter activity. P19 cells were co-transfected with *Oct4*(−1059)-Luc and various amounts of pcDNA3-EF1-α-*Meis1a*. After 24 h, luciferase activities and Meis1a expression levels were analyzed. *p<0.001 significantly different from vacant vector-introduced cells. *n = *3. (**C**) Schematic presentation of *Oct4* promoter-introduced luciferase reporters. *Oct4*(−1059)-Luc has four putative Meis1-BEs. (**D**) Functional analysis of Meis1-BEs. P19 cells were transfected with *Oct4*(−1059)-Luc, *Oct4*(−698)-Luc, *Oct4*(−506)-Luc, or *Oct4*(−254)-Luc together with 60 ng pcDNA3-EF1-α-*Meis1a*. After 24 h, luciferase activities were analyzed. *p<0.001 significantly different from *Oct4*(−698)-Luc, *Oct4*(−506)-Luc, and *Oct4*(−254) Luc. *n = *3. (**E**) Occupation of the *Oct4* promoter by Meis1a and HDAC1. Aggregated P19 cells were treated with RA for 0 and 6 h. Genomic chromatin fragments were immunoprecipitated with anti-Oct4 and anti-HDAC1 antibodies and DNAs were extracted. PCR was carried out using the primer set covering the −1062 to −778 region of the *Oct4* promoter. Aliquots of 10% antibody-untreated DNA samples were used for input DNA. (**F**) Quantification of the *Oct4* promoter-bound Meis1a and HDAC1 indicated in **E**. (**G**) Schematic presentation of the adjacent Meis1-BEs3/4 region. In this region, putative Pbx- and Hox-BEs also existed.

Oct4 expression in neural differentiation was reduced dependent on the increasing amount of Meis1a and disappeared when the maximal expression of Meis1a was observed (Fig. 1F–I). Therefore, it is likely that Meis1a represses Oct4 expression. To test this idea, P19 cells were transfected with vacant or *Meis1a* expression vectors and expression levels of Oct4 were analyzed by WB after 24 h. Regardless of treatment with or without RA, Oct4 expression was lower by the ectopic expression of *Meis1a* than that in vacant vector-introduced cells (Fig. 4A). To further analyze the suppressive effect of Meis1a on *Oct4* expression, we constructed luciferase reporter plasmids, which were inserted at −1059 to +225, −698 to +225, −506 to +225, and −254 to +225 prompter regions of the *Oct4* (*Pou5f1*) gene, and designated them as *Oct4*(−1059)-Luc, *Oct4*(−698)-Luc, *Oct4*(−506)-Luc, and *Oct4*(−254)-Luc, respectively. In P19 cells, *Oct4*(−1059)-Luc activity, which possesses four putative Meis1-binding elements (Meis1-BEs; consensus Oct4-BE; TGACAG), was suppressed by Meis1a in a dose-dependent manner (Fig. 4B, C). On the other hand, *Oct4*(−698)-Luc, *Oct4*(−506)-Luc, and *Oct4*(−254)-Luc activities, in which Meis1-BEs3/4 were deleted, were no longer suppressed by Meis1a (Fig. 4D), suggesting that putative Meis1-BEs3/4 are essential for the suppression of *Oct4* expression.

To examine whether Meis1a binds to putative Meis1-BEs3/4 in the *Oct4* promoter region, the ChIP assay was performed. In the undifferentiated state, putative Meis-BEs3/4-bound Meis1a was a negligible level (Fig. 4E, F). Interestingly, after 12 h of RA treatment, when Meis1a expression was substantially induced and Oct4 expression levels had just began to decrease, Meis1a was bound to putative Meis1-BEs3/4. Simultaneously, HDAC1 was detected in this region. It is noteworthy that Meis1 binding partner’s putative Pbx and Hox-BEs exist in the adjacent region of Meis1-BEs3/4 (Fig. 4G) [17]. Thus, these results imply that Meis1a down-regulates *Oct4* expression via direct binding to Meis1-BEs3/4 during 12–48 h of the late-early stage of neural differentiation.

### Meis1a Induces Large Neurospheres Accompanying Up-regulation of NSC and Neural Progenitor Markers

**Figure 5:**
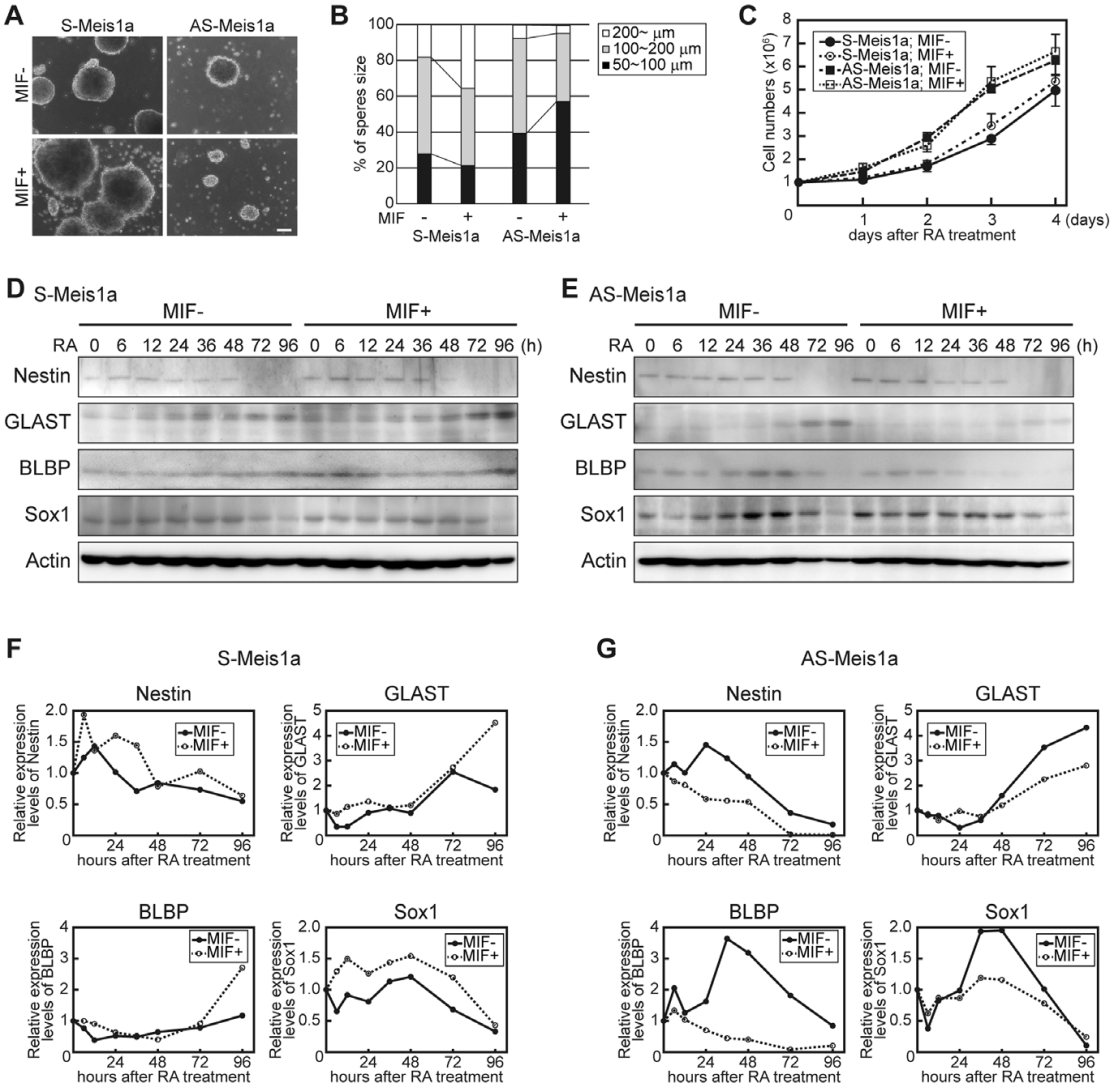
Stimulatory effect of Meis1a on the formation of neurospheres consisted of NSCs/neural progenitor cells. (**A**) Effect of Meis1a on sphere formation during neural differentiation. Aggregated S-Meis1a and AS-Meis1a were treated with RA in the presence or absence of MIF. After 4 days, spheres were analyzed under a phase-contrast microscope. *Scale bar* = 100 µm. (**B**) Quantification of sphere sizes of S-Meis1a and AS-Meis1a indicated in **A**. More than 400 spheres of each sample were analyzed. (**C**) Effect of Meis1a on the cell growth during neural differentiation. (**D** and **E**) Aggregated S-Meis1a and AS-Meis1a cells were treated with RA together with or without MIF. Effects of Meis1a on the NSC/neural progenitor marker expressions. Cell lysates from RA-primed S-Meis1a (**D**) and AS-Meis1a (**E**) cells with or without MIF were analyzed by WB with anti-Nestin, anti-GLAST, anti-BLBP and anti-Sox1 antibodies. (**F** and **G**) Quantification of expression levels of NSC/neural progenitor markers in S-Meis1a and AS-Meis1a cells as shown in **D** and **E**, respectively.

Since ectopic expression of *Meis1a* enhanced both neuronal and astrocyte differentiation of RA-primed P19 cells (Fig. 2), we analyzed the effect of *Meis1a* on the generation of free-floating aggregates called neurospheres, which mainly consist of NSCs and progenitor cells [28]. Aggregated S-Meis1a and AS-Meis1a cells were cultured in bacteriological grade plates in the presence of RA with or without MIF, and after 4 days the number and size of generated neurospheres were determined. Upon the addition of MIF, the fraction of large neurospheres (≥200 µm in diameter) in S-Meis1a cells was robustly higher than that in the MIF-untreated control, whereas the fraction of large neurospheres in AS-Meia1a cells was reduced (Fig. 5A, B). Nonetheless, the growth rate of AS-Meis1a cells was somewhat higher than that of S-Meis1a cells regardless of treatment with or without MIF (Fig. 5C), suggesting that in the presence of MIF, S-Meis1a cells maintain a higher number of NSCs/neural progenitor cells than that in AS-Meia1a cells.

Radial glial cells have been identified as a major source of neurons *in vivo* and i*n vitro*, and express the intermediate filament Nestin as well as NE cells [29]. It is well known that the astrocyte-specific glutamate transporter GLAST and BLBP are the radial glia markers [30], [31]. In addition, Sox1, one of the SoxB1 family transcription factors, maintains the undifferentiated state of cortical neural progenitors [32]. Based on these reports, we analyzed the expression levels of Nestin, GLAST, BLBP and Sox1 during the generation of neurospheres in S-Meis1a cells with or without MIF. Upon the addition of MIF, these markers were induced in S-Meis1a cells (Fig. 5D, F). On the other hand, upon the addition of MIF AS-Meis1a cells generated small neurospheres that expressing lower levels of the markers compared with those in MIF(−) control cells (Fig. 5E, G).

**Figure 6:**
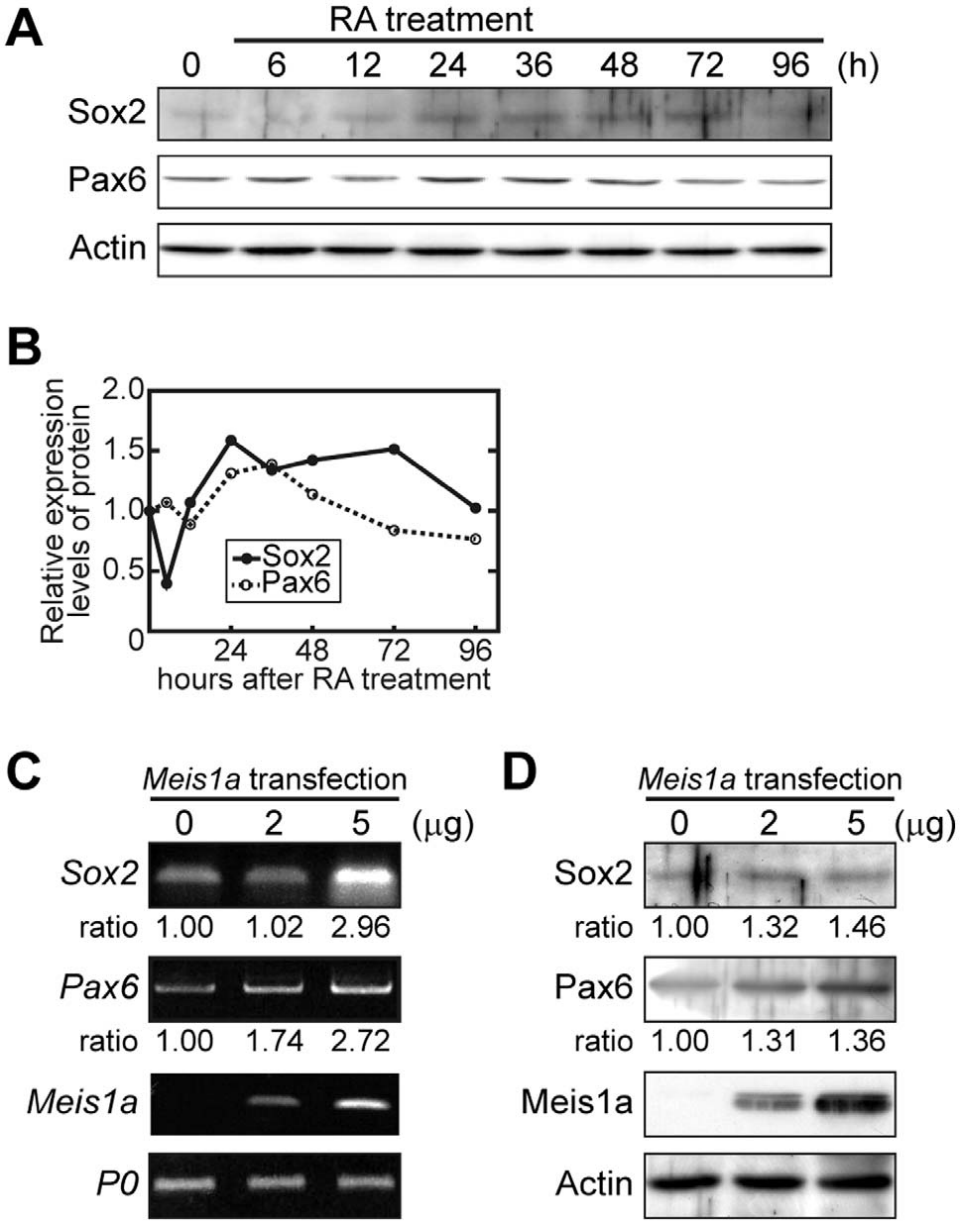
Effect of Meis1a on Sox2 and Pax6 expressions. (**A**) Expression patterns of Sox2 and Pax6 during neural differentiation. Aggregated P19 cells were treated with RA for various times and analyzed by WB with anti-Sox2 and Meis1 antibodies. (**B**) Quantification of expression levels of Sox2 and Pax6 as shown in **A**. (**C** and **D**) Stimulation of *Sox2* and *Pax6* mRNA and protein expressions by ectopic expression of Meis1a. Monolayer-cultured P19 cells were transfected with various amounts of pcDNA3-EF1-α-*Meis1a* and after 12 h, *Sox2* and *Pax6* mRNAs and proteins were analyzed by RT-PCR (**C**) and WB (**D**), respectively.

It has also been reported that Sox2, another SoxB1 family transcription factor, maintains neural progenitor cells in addition to the maintenance of pluripotent ES cells [32], and Pax6, a key transcription factor in the development of the central nervous system, drives NE to radial glia progression during the differentiation of mouse ES cells [33]. Therefore, at first, we analyzed the expression patterns of Sox2 and Pax6 during neural differentiation of RA-primed P19 cells. Both protein expressions were substantially enhanced after 24 h of RA addition (Fig. 6A, B). The appearance of the enhanced expression of both proteins was later than those of Oct4 and Meia1a (Fig. 1H, I). To test the idea that the up-regulation of Sox2 and Pax6 is triggered in the downstream of Meis1a signaling pathway, monolayer-cultured P19 cells were transfected with the *Meis1a* expression vector and after 12 h *Sox2* and *Pax6* expression levels were analyzed by RT-PCR and WB. mRNA and protein expression levels of *Sox2* and *Pax6* were dose-dependently enhanced by the transient expression of Meis1a (Fig. 6C, D), suggesting that Sox2 and Pax6 are involved in the generation of neurospheres consisting of NSCs and neural progenitor cells downstream of the Meis1a signaling pathway.

## Discussion

In this study, based on the analysis of *Oct4* and *Meis1* promoter activities, we found the possibility that up-regulated Oct4 during 12 h of the immediate-early stages of RA-primed P19 EC cell neural differentiation dose-dependently stimulates *Meis1a* gene expression accompanying AcH3 and appearance of 5hmC (Fig. 3), while the Oct4-induced Meis1a suppresses *Oct4* expression via direct binding to the distal Meis1a-BEs3/4 of the *Oct4* promoter together with HDAC1 (Fig. 4). These data provide the first insight into the molecular events underlying the involvement of direct crosstalk between Oct4 and Meis1a on mutual gene expression in neural fate choice.

Oct4, Sox2, and Nanog that cooperatively maintain ES cell identity via suppression of developmentally important transcription factors including Meis1, Pax6, Otx1, and HoxB1, also orchestrate germ layer fate selection [34]. Thomson et al. reported that Oct4 suppresses NE differentiation and promotes ME differentiation from mouse ES cells, while Sox2 inhibits ME differentiation and promotes NE differentiation, indicating that asymmetric regulation of Oct4 and Sox2 determines cell fate choice [1]. Nonetheless, in neural differentiation of RA-primed P19 cells, we previously observed that *Oct4, cyclophylin A, RARα* and *Otx2* gene expressions were substantially stimulated within 1 h of RA addition and reached their maximal levels after 6 h [23]. Subsequently, Oct4 expression was reduced to original levels at ∼ 24 h and was undetectable after 48 h. The expression of *Otx2*, a NE marker, vanished completely by 12 h. Conversely, COUP-TFI, a repressor of the *Oct4* gene, was enhanced during 12–48 h of the late-early stages [23], [35]. The RAR/RXR heterodimer activated by cyclophylin A specifically bound to RARE*oct* (−48 to −28) in the *Oct4* proximal promoter region and activated Oct4 gene expression [8], [9], [23]. Since both COUP-TFI and RAR/RXR regulate *Oct4* gene expression via RARE*oct,* it seems that RAR/RXR promotes *Oct4* expression in the immediate-early stages, whereas COUP-TFI represses in the late-early stages. The discrepancy in *Oct4* mRNA and protein expression patterns in the neural fate decision among our results and those of other reports can be reconciled in a model in which the temporal up-regulation of Oct4 is required at the immediate-early stages of neural differentiation [1], [15].

Liang et al. reported that Oct4 and Nanog interact with each other and proteins from multiple repressor complexes including NuRD (Mi-2/nucleosome remodeling deacetylase) that include chromodomain helicase CDH3/4, deacelase HDAC1/2, 5mCpG-binding proteins Mbd3 and Mta1, and a similar complex that lacks Mbd3, NODE (Nanog and Oct4 associated deacetylase) [26]. Depletion of Mta1 de-represses genes related to endoderm differentiation such as GATA6 and FoxA2, indicating that an intact NODE complex is required for suppression of premature cell lineage commitment [26]. Moreover, Sérandour et al. recently reported that 5hmC generated form 5mC by dioxygenase Tet is associated with genes expressed in neural differentiation of P19 cells and in adipocyte differentiation of 3T3 cells [27]. Distal promoter regions of *Meis1* gaining 5hmC together with H3Km2 and H3K27ac in P19 cells behave as differentiation-dependent transcriptional enhancer [27]. In this study, we observed that in the undifferentiated P19 cells, Oct4 existed in the putative Oct4-BEs1/2 of *Meis1* together with HDAC1, while in the immediate-early neural differentiation stage, Oct4 occupied the Oct4-BEs1/2 together with the significant levels of AcH3 and 5hmC (Fig. 3). Summing up our present data and other reports, it is highly possible that depending upon the neural differentiation cues such as cell aggregation and RA, Oct4-associated repressor complexes can be converted to transcriptional activator complexes. Therefore, only 1.2-fold transient induction of Oct4 in the immediate-early stage and the qualitative change of Oct4- containing transcription complex are thought to be enough to induce *Meis1* gene expression. Nonetheless, it is presently unclear how Oct4-containing transcription complex induces 5hmCpG from 5mCpG.

Signal transduction pathways play diverse, context-dependent roles in development. Neural differentiation of P19 cells is dependent on cell aggregation by which Wnt-1 is up-regulated [36]. Ectopic expression of Wnt-1 directs P19 cells to differentiate into neurons, but not astrocytes, through the canonical Wnt pathway [37]. On the other hand, in human NT2 EC cells RA induces early neuronal differentiation via induction of two noncanonical Wnt-4 and Wnt-11, which suppress the canonical pathway [38]. Thus, the idea that inhibition of β-catenin/Tcf activity is essential for neuronal differentiation may appear to contradict reports showing that β-catenin activity is involved in the neurogenic process [37], [39]–[41]. This discrepancy can also be resolved in a model in which β-catenin/Tcf activity is required at the late-early stages of neural differentiation.

In this study, we observed that expression levels of Sox2 and Pax6, which function to maintain neural progenitor cells and differentiate radial glia cells from NE, respectively, were substantially induced 24 h after RA addition [32], [33]. These transcription factors could be also induced by ectopic and transient expression of *Meis1a* in monolayer-cultured P19 cells (Fig. 6). Furthermore, Davidson et al. have recently shown that Wnt/β-catenin signaling is repressed by Oct4 [42]. Although we cannot rule out the possible involvement of nitric oxide synthase NOS3 and microRNAs at present [43], [44], taking into account our present results and those of other reports, we propose the idea that the transient up-regulation of Oct4 together with qualitative change of Oct4-containing transcription complex within 12 h of the immediate-early stages by RAR/RXR via RARE*oct* triggers the neural fate decision, and subsequently in the late-early stages, at least in part, Oct4-induced Meia1a and COUP-TFI synergistically suppress Oct4 expression via the distal Meis1-BEs and RARE*oct*, respectively.

Meis family proteins are required for Pbx/Hox complexes to exert positive or negative transcriptional control [45]. Consistent with this observation, Meis family proteins cooperate with Pbx and Hox for hind brain patterning [18]–[20]. Huang et al. proposed that within certain cell contexts, compacted chromatin in enhancer/promoter regions and/or the more dominant activity of the corepressor function associated with Pbx repression domains cause transcriptional inactivation despite the presence of Hox and Meis possessing transcriptional activation domains [25]. This is reversed by cellular signaling such RA and protein kinase A (PKA) and their downstream effectors. PKA could induce the recruitment of coactivator associated Hox and Meis activation domains or inhibit the HDAC activity of the corepressors associated with Pbx. A net positive output would result from predominant coactivator over corepressor activity. These events lead to a shift from transcriptional silencing to activation. We found that putative Meis1-BEs3/4 (−1028 to −1015) in the *Oct4* promoter region were required for the suppression of *Oct4* promoter activity by Meis1a (Fig. 4). In this adjacent region, putative Pbx and Hox-BEs were also found (Fig. 4). Moreover, ChIP analysis showed that Meis1a and HDAC1 could bind to putative Meis1-BEs3/4. Thus, it seems that during 12–48 h of the late-early stages of neural differentiation, the Meis1a/Pbx/Hox heterotrimer suppresses *Oct4* gene expression via the recruitment of HDAC1.

The *Hoxb1* autoregulatory element (ARE) has been precisely analyzed. The Hoxb1 ARE is a Pbx/Hox complex target that directs expression of rhombomere 4 (r4) in the developing hindbrain [46]. It contains binding sites for the Pbx/Hox complex, Meis/Prep1, Sox, and Oct transcription factors, although only Pbx/Hox sites are required for r4 enhancer function. Hoxb1 ARE drives the expression of a lacZ reporter in P19 cells induced to differentiate the neural pathway by aggregation in the presence of RA, whereas P19 cell monolayers fail to activate ARE following RA addition [45]. Nonetheless, the promoter region of the *Meis1* gene has not been fully analyzed yet. In this study, we found that Oct4 could dose-dependently activate Meis1 promoter activity via direct binding to putative Oct4-BEs1/2. To our knowledge, this is the first demonstration of Oct4-dependent stimulation of *Meis1a* gene expression in the immediate-early stages of neural differentiation.

NSCs are able to generate clonal structures, neurospheres, that exhibit intra-clonal neural cell-lineage diversities; i.e., they contain, in addition to NSCs, neuronal and glial progenitors in different states of differentiation [47]. Chiasson et al. have reported that the ependymal cells can proliferate *in vitro* to form small neurospheres that do not have the ability to self-renew and only produce GFAP-positive glial cells, while subependymal cells can form large spheres having the self-renewing and multipotential characteristics of NSCs [48]. In this study, large spheres induced by ectopic expression of sense *Meis1a* RNA formed were highly expressed the NSC/neural progenitor markers and efficiently generated β-tubulin (III)-positive neurons and GFAP/S100β-positive astrocytes (Figs. 2 and 5). In addition, ectopically temporal expression of Meis1a in monolayer-cultured P19 cells could induce the expression of Sox2 and Pax6 (Fig. 6), which maintains neural progenitors in the some context [32], [49]. Pax6 also controls radial glial cell differentiation, which has been identified as a major source of neurons during development [29], [33], [50]. Therefore, it seems likely that Oct4-up-regulated Meis1a rapidly drives the division of neural progenitors from NSCs together with sustained maintenance of core NSCs and forms large neurospheres, which lead to efficient both neuronal and astrocyte differentiations.

Since P19 EC cells possess many properties similar to ES cells established from mice and humans [51], it may be possible that the expression system of Meis1a can be utilized for the production of neurons from human ES cells in combination with the PRP19α expression system 23,52.
